# Role of Platelet Glycoprotein VI and Tyrosine Kinase Syk in Thrombus Formation on Collagen-Like Surfaces

**DOI:** 10.3390/ijms20112788

**Published:** 2019-06-07

**Authors:** Natalie J. Jooss, Ilaria De Simone, Isabella Provenzale, Delia I. Fernández, Sanne L.N. Brouns, Richard W. Farndale, Yvonne M.C. Henskens, Marijke J.E. Kuijpers, Hugo ten Cate, Paola E.J. van der Meijden, Rachel Cavill, Johan W.M. Heemskerk

**Affiliations:** 1 Department of Biochemistry, Cardiovascular Research Institute Maastricht (CARIM), Maastricht University, 6229 ER Maastricht, The Netherlands; n.jooss@maastrichtuniversity.nl (N.J.J.); i.desimone@maastrichtuniversity.nl (I.D.S.); i.provenzale@maastrichtuniversity.nl (I.P.); d.fernandezdelafuent@maastrichtuniversity.nl (D.I.F.); sanne.brouns@maastrichtuniversity.nl (S.L.N.B.); marijke.kuijpers@maastrichtuniversity.nl (M.J.E.K.); h.tencate@maastrichtuniversity.nl (H.t.C.); p.vandermeijden@maastrichtuniversity.nl (P.E.J.v.d.M.); 2 Department of Biochemistry, University of Cambridge, Cambridge CB2 1QW, UK; rwf10@cam.ac.uk; 3 Laboratory for Clinical Thrombosis and Hemostasis, Maastricht University Medical Center, 6229 ER Maastricht, The Netherlands; yvonne.henskens@mumc.nl; 4 Department of Internal Medicine, Cardiovascular Research Institute Maastricht (CARIM), Maastricht University, 6229 ER Maastricht, The Netherlands; 5 Department of Data Science and Knowledge Engineering, Cardiovascular Research Institute Maastricht (CARIM), Maastricht University, 6229 ER Maastricht, The Netherlands; rachel.cavill@maastrichtuniversity.nl

**Keywords:** calcium, collagen, glycoprotein VI, platelet activation, protein tyrosine kinase, thrombus

## Abstract

Platelet interaction with collagens, via von Willebrand factor, is a potent trigger of shear-dependent thrombus formation mediated by subsequent engagement of the signaling collagen receptor glycoprotein (GP)VI, enforced by integrin α_2_β_1_. Protein tyrosine kinase Syk is central in the GPVI-induced signaling pathway, leading to elevated cytosolic Ca^2+^. We aimed to determine the Syk-mediated thrombogenic activity of several collagen peptides and (fibrillar) type I and III collagens. High-shear perfusion of blood over microspots of these substances resulted in thrombus formation, which was assessed by eight parameters and was indicative of platelet adhesion, activation, aggregation, and contraction, which were affected by the Syk inhibitor PRT-060318. In platelet suspensions, only collagen peptides containing the consensus GPVI-activating sequence (GPO)_n_ and Horm-type collagen evoked Syk-dependent Ca^2+^ rises. In whole blood under flow, Syk inhibition suppressed platelet activation and aggregation parameters for the collagen peptides with or without a (GPO)_n_ sequence and for all of the collagens. Prediction models based on a regression analysis indicated a mixed role of GPVI in thrombus formation on fibrillar collagens, which was abolished by Syk inhibition. Together, these findings indicate that GPVI-dependent signaling through Syk supports platelet activation in thrombus formation on collagen-like structures regardless of the presence of a (GPO)_n_ sequence.

## 1. Introduction

Platelet interaction with subendothelial collagen is a crucial step in hemostasis or arterial thrombosis after vascular injury or rupture of an atherosclerotic plaque, respectively [1,2]. In blood flowing with high shear rates, the initial capture of platelets is mediated by the von Willebrand factor (VWF), which avidly binds to collagens and is a ligand for the glycoprotein (GP) complex GPIb–V–IX [3]. The two platelet collagen receptors integrin α_2_β_1_ and GPVI ensure stable platelet adhesion and mediate platelet activation [4,5]. For over 20 years, GPVI has been recognized as the central signaling collagen receptor on platelets [6,7].

Studies using genetically modified mice have shown that the (patho)physiological process of arterial thrombus formation can be approximated using microfluidics devices, in which whole blood is perfused over a collagen surface [8]. The collagen fibers immobilized in such devices, for instance those applied as microspots, also bind plasma VWF and thus promote shear-dependent adhesion, activation, and aggregation of platelets [3,9]. Previous results have revealed a strong interplay of GPIb-V-IX, GPVI, and integrin α_2_β_1_ in the formation of large and stable multilayered thrombi in a way where the two other receptors enforce the GPVI-induced activation of platelets [10,11]. Markedly, the thrombi that formed on collagen fibers appeared to be heterogeneous in structure, with on the one hand patches of aggregated platelets with activated integrin α_IIb_β_3_ binding fibrinogen and CD62P expression via α-granule secretion and on the other hand single procoagulant platelets, exposing phosphatidylserine (PS), which is required for coagulation factor binding [12]. Particularly active in supporting thrombus formation is the standard collagen preparation “Horm” (collagen-H), which is a fibrillar type I collagen prepared in a proprietary process that is commonly used for diagnostics in the clinical laboratory. Still unexplained is why other fibril-forming type I and III collagen preparations, also binding with VWF, are less active in supporting thrombus formation under flow [9,13].

In previous years, a number of synthetic collagen-derived triple-helical peptides have been identified that, similarly to collagen-H, bind to GPVI and/or integrin α_2_β_1_ and thus induce platelet adhesion and activation in vitro [10]. Peptides containing the (GPO)_n_ motif, in contrast to the supposedly inactive (GPP)_n_ motif, bind to GPVI, while peptides with the GFOGER motif act as strong ligands for integrin α_2_β_1_ [14,15,16]. The prototypes of such triple-helical peptides are the cross-linked collagen-related peptide (CRP-XL) with a (GPO)_10_ sequence, which hence acts as a potent GPVI agonist, and the combined GFOGER–(GPO)_n_ sequence, which binds to platelets via both receptors. Subtle changes in the GFOGER sequence have been found to alter the affinity for α_2_β_1_. For instance, the substitution of phenylalanine in GFOGER by alanine in GAOGER resulted in lower-affinity α_2_β_1_ binding and in diminished platelet adhesion under static conditions [17].

Platelet activation through GPVI [18,19,20], but not via GPIb-V-IX [21], relies on a potent protein tyrosine kinase cascade, culminating in the activation of the tyrosine kinase Syk. This GPVI signaling pathway involves phosphorylation of the Fc receptor γ-chain via Src-family kinases and construction of a GPVI signalosome, in which Syk phosphorylates and activates phospholipase C (PLC)-γ2, causing a rise in the central second messenger, Ca^2+^ [18,22,23,24]. However, the relative importance of this pathway has not been investigated thus far in platelets interacting under flow with surface-immobilized collagen peptides or fibrillar collagens.

In the present paper, we aimed to investigate the role of the GPVI-Syk pathway in thrombus formation on collagen-like surfaces at a high shear rate. In particular, we assessed the subprocesses of platelet adhesion, aggregation, and contraction as well as specific platelet activation responses. For this purpose, we used a panel of collagens, collagen peptides, and collagen-H (with established GPVI dependency) and the selective Syk inhibitor PRT-060318 (Syk-IN). The latter compound has recently been used to specify Syk-dependent pathways in mouse platelets [21,25] and in human T cells [26]. As a direct readout of this signaling pathway, we also assessed Syk-dependent rises in cytosolic Ca^2+^.

## 2. Results

### 2.1. GPVI-Dependent and Syk-Dependent Platelet Activation by Collagen Peptides

As a first estimation of the potency of distinct collagen peptides to act as ligands for platelet GPVI, we examined their ability to stimulate PLCγ2-mediated rises in cytosolic Ca^2+^ using Fura-2-loaded platelets. For a selective inhibitor of the GPVI pathway, we used the compound PRT-060318 (Syk-IN), which was earlier used to identify Syk-dependent activation processes in platelets mediated by GPVI [21,25] or CLEC2 [27]. The Syk-IN compound was found to phenocopy the effects of Syk depletion on platelet responses in *Syk*^−/−^ bone marrow chimeric mice [28]. Moreover, in human platelets, Syk-IN selectively blocked the GPVI/Syk-dependent tyrosine phosphorylation and aggregation responses induced by fibrin [29].

To confirm the selectivity of Syk-IN as an inhibitor of GPVI-induced responses of human platelets, we monitored its effect (using 5 µM throughout) on the aggregation of platelets induced by CRP-XL, thrombin, or stable ADP. This inhibitor caused the complete inhibition of aggregation only with the GPVI agonist CRP-XL, whereas with thrombin or ADP it was ineffective (Figure S1A). This was in agreement with earlier studies performed with mouse platelets [21,25]. Additional control experiments with Fura-2-loaded human platelets indicated that Syk-IN did not suppress thrombin- or ADP-induced Ca^2+^ rises (Figure S1B).

**Figure 1:**
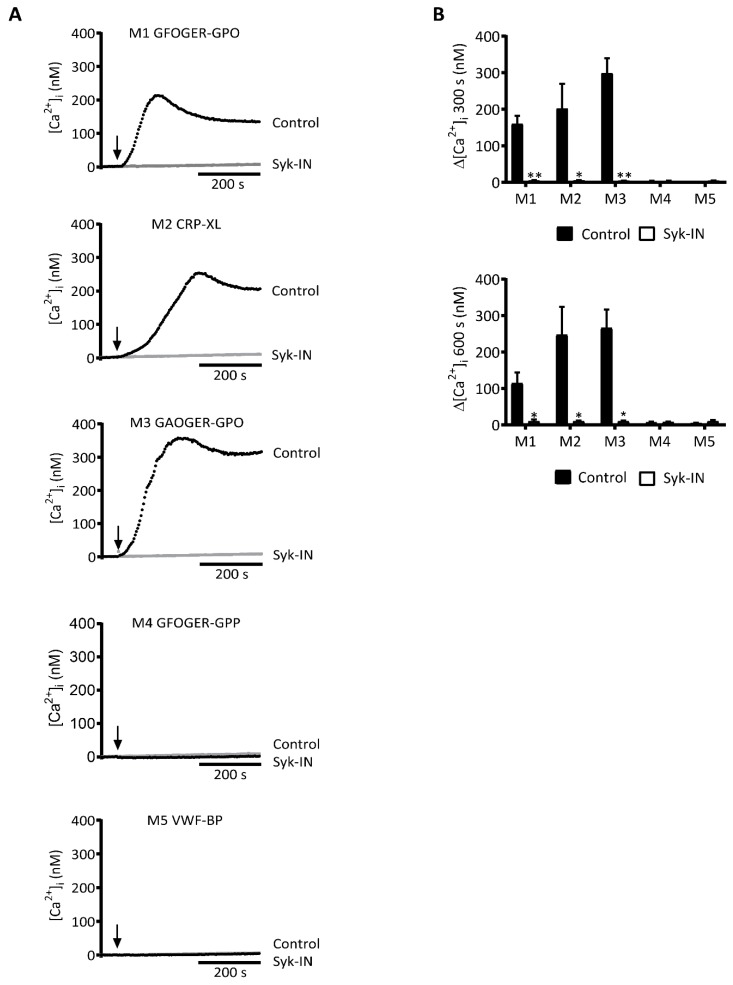
Syk inhibition affecting platelet Ca^2+^ rises by collagen peptides with (GPO)_n_ motif. Fura-2-loaded platelets in 96-well plates were pre-incubated with Syk-IN (5 µM) or left untreated before stimulation with collagen peptide (M1-5, 10 µg/mL). Changes in [Ca^2+^]_i_ were recorded over time per well-plate row by ratio fluorometry using a FlexStation 3. Peptides were injected into wells at 60 s (arrow) and reached platelets in a diffusion-limited way. (**A**) Calibrated [Ca^2+^]_i_ traces recorded over 600 s in the absence (black, control) or presence (gray) of a Syk inhibitor. Traces are representative of three experiments. (**B**) Quantification for M1-5 of increased [Ca^2+^]_i_ at 300 s (top graph) or 600 s (bottom graph). Means ± SEM (*n* = 3). Paired Student’s *t*-tests; * *p* < 0.05, ** *p* < 0.01.

Using Syk-IN, we then evaluated the role of Syk in platelet Ca^2+^ fluxes, which were induced by several collagen peptides presumed to be GPVI-dependent or -independent. With three peptides containing the consensus GPVI-activating motif (GPO)_n_, i.e., GFOGER-GPO (for convenience designated as M1, see Table 1), CRP-XL (M2), and GAOGER-GPO (M3), we observed a potent rise in [Ca^2+^]_i_, which was fully abolished in the presence of Syk-IN (Figure 1). Close examination of the Ca^2+^ traces showed some differences between M1-3 at onset and the maximum value. The reason for these differences was unclear, but they may have been linked to variations in the peptide conformation or GPVI clustering capacity of the triple-helical peptide in question. On the other hand, two other collagen peptides containing a (GPP)_n_ motif instead of (GPO)_n_ were unable to induce [Ca^2+^]_i_ rises: These were GFOGER-GPP (M4) and the VWF-binding peptide (M5). These Ca^2+^ traces were not influenced by the presence of Syk-IN. Overall, these results indicated a high Syk dependency of the platelet [Ca^2+^]_i_ rises, which were induced by the (GPO)_n_-containing collagen peptides containing an established GPVI-activating sequence.

### 2.2. GPVI- and Syk-Dependent Parameters of Thrombus Formation on Collagen Peptides

To assess how the five collagen peptides supported whole-blood thrombus formation, we applied these as microspots (M1–5) in a microfluidic device, as described before [9]. The microspots were supplemented with VWF-BP (peptide binding plasma VWF) to allow for GPIb-V-IX-mediated trapping of platelets. Whole-blood perfusion was performed at a wall-shear rate of 1000 s^−1^. Through end-stage (3.5 min) multicolor microscopic imaging, it was possible to analyze up to eight thrombus and platelet characteristics: Overall platelet deposition (parameter P1, see Table 1); platelet aggregation (P2); thrombus signature, i.e., morphology, multilayers, and contraction (P3-5); platelet procoagulant activity, measured as PS exposure (P6); platelet activation parameter CD62P expression (P7); and fibrinogen binding to activated integrin α_IIb_β_3_ (P8).

**Figure 2:**
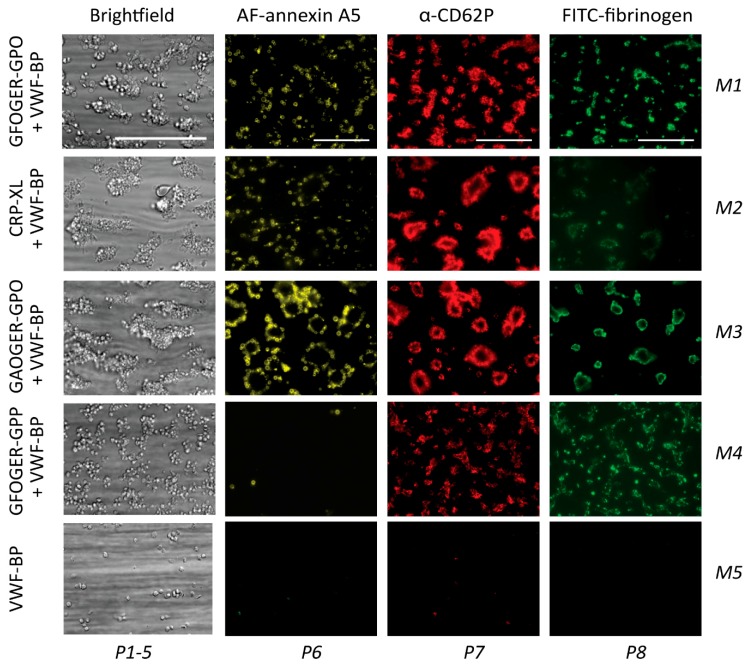
Thrombus formation on immobilized collagen peptides with or without a (GPO)_n_ motif. Whole blood was perfused over microspots M1 (GFOGER-GPO + VWF-BP), M2 (CRP-XL + VWF-BP), M3 (GAOGER-GPO + VWF-BP), M4 (GFOGER-GPP + VWF-BP), and M5 (VWF-BP), with assumed platelet adhesion via GPIb, GPVI, and/or integrin α_2_β_1_, as in Table 1. The wall-shear rate was 1000 s^−1^, with a perfusion time of 3.5 min. Representative bright-field microscopic images at the end stage are shown for an analysis of platelet deposition (parameter P1) and thrombus characteristics (P2–5). In addition, end-stage three-color fluorescence images for an analysis of PS exposure (AF568 annexin A5, P6), CD62P expression (AF647 α-CD62P, P7), and fibrinogen binding (FITC, P8) are shown. Scale bars represent 50 μm.

**Figure 3:**
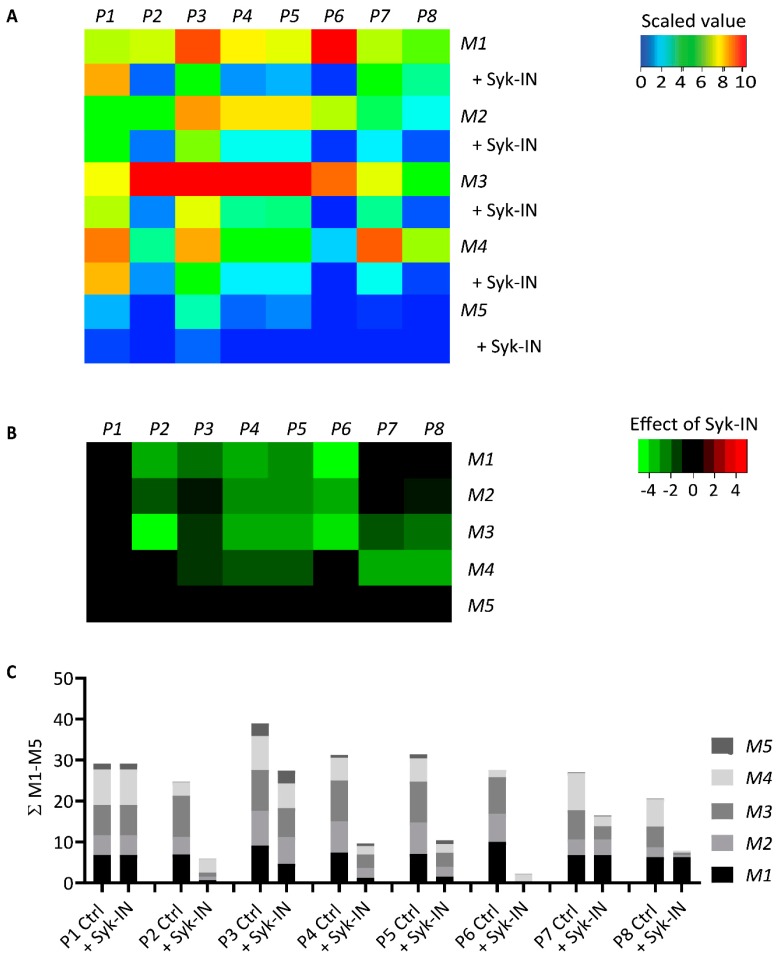
Effect of Syk inhibition on parameters of thrombus formation on immobilized collagen peptides. Blood samples pre-incubated with vehicle (Ctrl) or Syk-IN (20 µM) flowed over microspots M1–5, and the thrombi formed were imaged to obtain parameters P1–8, as in Figure 2. The effects of Syk-IN were assessed per blood sample, surface, and parameter. Mean values from individual blood samples (*n* = 5–7) were univariate-scaled to 0–10 per parameter across all surfaces M1–9. (**A**) Heatmap of scaled parameters, demonstrating the mean effects of Syk-IN. The rainbow color code indicates scaled values between 0 (blue) and 10 (red). (**B**) Subtraction heatmap representing the scaled effects of Syk-IN, filtered for relevant changes (*p* < 0.05, paired Student’s *t*-test per surface and parameter). The color code represents decreases (green) or increases (red) in comparison to control runs. (**C**) Cumulative inhibitory effect per parameter over all microspots, indicating relevant changes.

Typically, the collagen peptides containing (GPO)_n_ (M1–3) produced large thrombi with aggregated platelets with high levels of activation markers, i.e., PS exposure, CD62P expression, and integrin activation (Figure 2). In contrast, the non-GPVI-stimulating (GPP)_n_ peptide GFOGER-GPP (M4) caused the formation of smaller thrombi with residual CD62P expression and integrin activation, but essentially no PS exposure. Quantification of the raw image data confirmed high parameter values for all (GPO)_n_ microspots from M1–3, indicating a strong ability to support thrombus formation (Figure S2). Interestingly, when comparing the two GFOGER peptides with or without the GPVI-binding motif (M1 or M4), the latter still induced residual platelet activation, in spite of lower thrombus signature scores (P4-5) and limited PS exposure (P6). Furthermore, M1 (GFOGER), which had a supposedly higher-affinity α_2_β_1_ binding motif than M3 (GAOGER), was less effective in promoting almost all thrombus parameters (P1,2,3–5,7,8). The differences between microspots were visualized in a univariate scaled heatmap of all parameters (Figure 3A). Together, the data suggested that the earlier distinction made between high- and low-affinity α_2_β_1_ binding sites (established under static conditions [11,15]) became confusing in part when immobilized collagen peptides were exposed to platelets in flowing whole blood. On the other hand, the apparent lack of both GPVI and α_2_β_1_ binding sites, as with M5, resulted in almost no stable platelet adhesion and activation.

Parallel flow runs on all microspots from M1–5 were performed with blood samples pretreated with Syk-IN (maximum effective dose of 20 µM) or DMSO vehicle. This resulted in marked reductions in the majority of thrombus parameters (Figure 3A). A subtraction heatmap pinpointing only relevant changes (*p* < 0.05) indicated that for M1–4, essentially all parameters except for P1 (platelet deposition) were reduced by Syk inhibition (Figure 3B). Most drastic complete reductions were seen with PS exposure (P6) on the “active” (GPO)_n_ surfaces of M1–3. Surprisingly, Syk inhibition also affected platelet activation at the supposedly non-GPVI (GPP)_n_ surface of M4. The other microspot, M5, was inactive in the absence of Syk-IN.

A summative plot was made indicating how individual (scaled) parameters were changed by Syk inhibition across all microspots (Figure 3C). This revealed a complete reduction in P6 (PS exposure), along with strong reductions in P2 (aggregate coverage), P4 (thrombus multilayer), P5 (thrombus contraction), and P8 (fibrinogen binding). Less affected were P3 (thrombus morphology) and P7 (CD62P expression).

### 2.3. GPVI-Induced and Syk-Dependent Platelet Activation by Different Collagens

**Figure 4:**
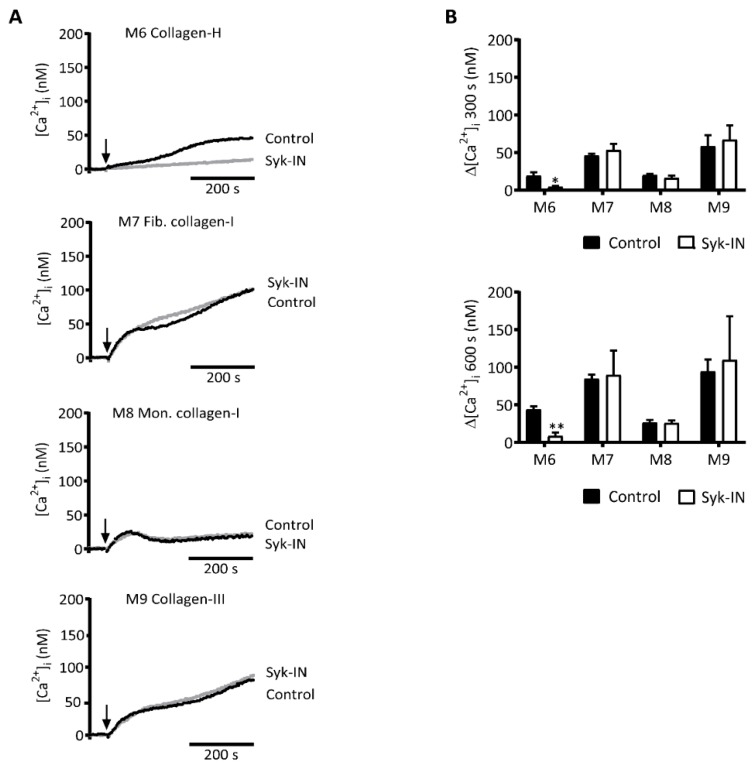
Syk inhibition differently affecting platelet Ca^2+^ rises by collagens. Fura-2-loaded platelets in 96-well plates were pre-incubated with Syk-IN (5 µM) or were left untreated before stimulation with different collagens (M6–9, 10 µg/mL). Changes in [Ca^2+^]_i_ were continuously monitored per well-plate row by ratio fluorometry using a FlexStation 3. Collagens were injected at 60 s (arrow), and they reached platelets in a diffusion-limited way. (**A**) Calibrated [Ca^2+^]_i_ traces recorded for 600 s in the absence (black, control) or presence (gray) of a Syk inhibitor. Traces are representative of three experiments. (**B**) Quantification of [Ca^2+^]_i_ rises after 300 s (top graph) and 600 s (bottom graph) for M1–5. Means ± SEM (*n* = 3). Paired Student’s *t*-tests; * *p* < 0.05, ** *p* < 0.01.

Subendothelial collagen types I and III are considered to be the major platelet-activating collagens in the vessel wall, acting via GPVI and α_2_β_1_ [30]. Equine standard collagen (collagen-H), likely a modified type I collagen, is the most commonly used collagen in studies of GPVI-induced platelet activation. This prompted us to compare four collagen preparations for their ability to support the GPVI-PLCγ2-Ca^2+^ activation pathway: The fibrous collagen-H (M6), human fibrillar collagen-I (M7), a degraded collagen-I (M8), and human fibrillar collagen-III (M9). Realizing that the very high molecular mass of collagens results in a heterogeneous interaction with platelets in suspension, we evaluated the [Ca^2+^]_i_ rises induced by these collagens. Markedly, the four collagens (M6–9) evoked a biphasic rise in [Ca^2+^]_i_, with an initial plateau level and a later second phase that was highest for M7 and M9 (Figure 4A,B). In absolute levels, the rises in [Ca^2+^]_i_ obtained with M6, 7, and 9 at a late time point of 600 s were 2–3-fold lower than those seen with the (GPO)_n_-containing collagen peptides (Figure 4 vs. Figure 1). This difference was likely due to the high molecular mass of the fibrillar-type collagens, which slowed down the rate and extent of diffusion-limited interactions with platelets, but it was also likely due to the higher density of the activation motif within the peptides. In addition, it appeared that Syk inhibition completely suppressed the [Ca^2+^]_i_ transients induced by the standard collagen-H (M6), but it did not alter the transients of other collagens (Figure 4). In the presence of indomethacin (10 μM, a thromboxane A_2_ pathway inhibitor), AR-C69931MX (10 μM, a P2Y_12_ receptor inhibitor), or MRS2179 (100 μM, a P2Y_1_ receptor inhibitor), the rises in [Ca^2+^]_i_ with collagens I–III were suppressed by 15–28%, 31–32%, or 17–31%, respectively, in a nonredundant way (data not shown). Taken together, this suggested the presence of a Syk-independent pathway for Ca^2+^ mobilization of suspended natural collagens, which in part came from autocrine activation mechanisms.

### 2.4. GPVI- and Syk-Dependent Platelet Responses in Thrombus Formation on Collagens

**Figure 5:**
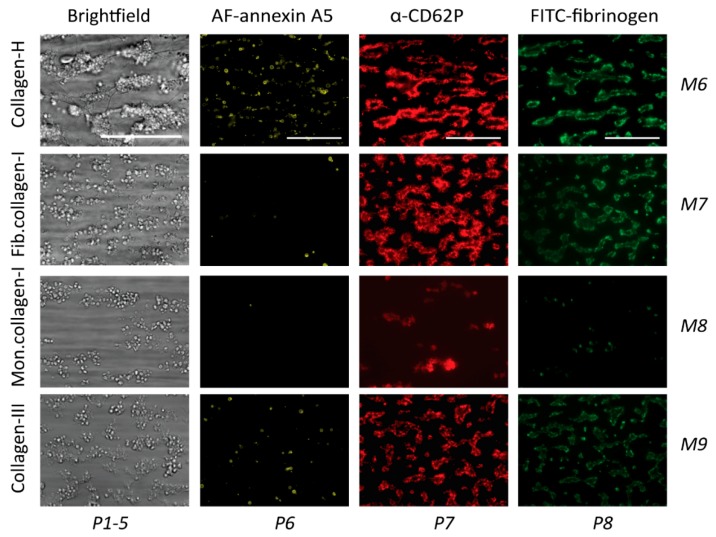
Thrombus formation on immobilized collagens. Whole blood was perfused over microspots M6 (collagen-H), M7 (fibrillar collagen-I), M8 (degraded collagen-I), and M9 (collagen-III). The wall-shear rate was 1000 s^−1^, and the perfusion time was 3.5 min. Representative bright-field microscopic images at the end stage are shown for an analysis of platelet deposition (parameter P1) and thrombus characteristics (P2–5). In addition, end-stage three-color fluorescence images for an analysis of PS exposure (AF568 annexin A5, P6), CD62P expression (AF647 α-CD62P, P7), and fibrinogen binding (FITC, P8) are shown. Scale bars represent 50 μm.

The same collagen preparations (M6–9) were also applied as microspots to test their ability to support thrombus formation under flow. As indicated in Figure 5, collagen-H (M6) was the most potent in provoking the formation of large aggregates of platelets with high PS exposure, granule secretion, and fibrinogen binding, in agreement with the known high GPVI- and α_2_β_1_-activating potency of this collagen when immobilized [9,11,12]. In comparison, the fibrillar type I (M7) and III (M9) collagens formed only small aggregates of platelets with remaining secretion and fibrinogen binding, with M9 causing residual PS exposure (Figure 5). The degraded collagen-I (M8) caused mostly single-platelet adhesion with incidental small-sized aggregates. The same information was obtained from the raw mean values of individual parameters with these surfaces (Figure S3).

**Figure 6:**
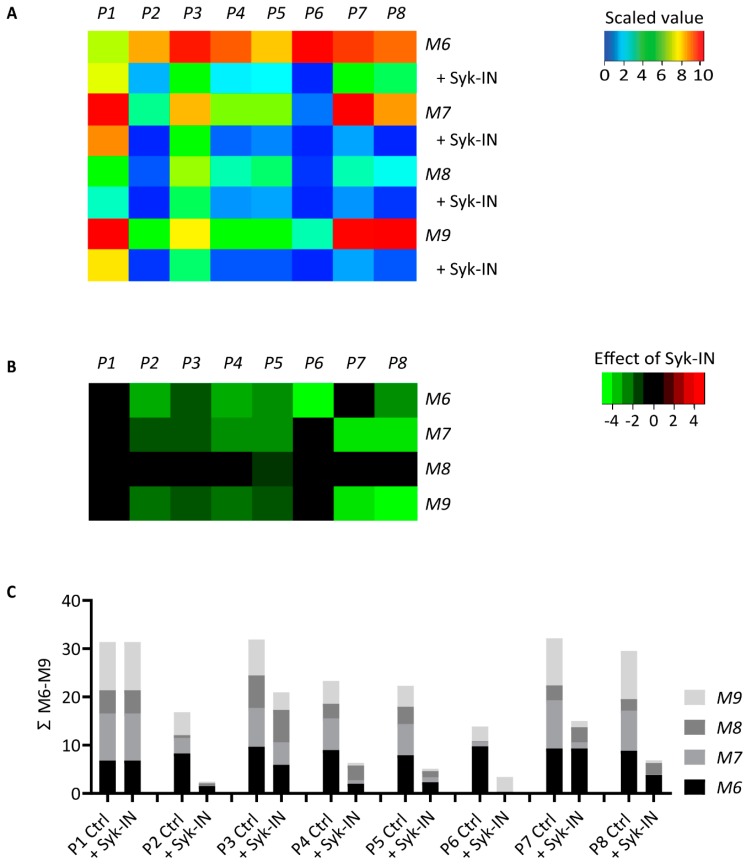
Effect of Syk inhibition on parameters of thrombus formation on immobilized collagen. Whole blood pre-incubated with vehicle (Ctrl) or Syk-IN (20 µM) was perfused over microspots M6–9, and thrombus formation was imaged to obtain parameters P1–8, as in Figure 5. The effects of Syk-IN were calculated per blood sample, surface, and parameter. Mean values for all blood samples (*n* = 5–7) were univariate-scaled to 0–10 per parameter across all surfaces of M1–9. (**A**) A heatmap of the scaled parameters showing the mean effects of Syk-IN. The rainbow color code gives scaled values between 0 (blue) and 10 (red). (**B**) A subtraction heatmap representing the scaled effects of Syk-IN, filtered for relevant changes (*p* < 0.05, paired Student’s *t*-tests per surface and parameter). The color code represents decreases (green) or increases (red) in comparison to control runs. (**C**) Cumulative inhibitory effect over all microspots per parameter, indicating relevant changes from control runs.

Heatmapping of the eight scaled parameter values confirmed that overall microspot thrombogenicity decreased in the order of M6 > M7,9 > M8 (Figure 6A). Treatment of the blood with Syk-IN left platelet deposition (P1) unchanged, but it decreased the thrombus signature and the platelet activation parameters (P2–5; P7,8) for several collagens. A subtraction heatmap was built with a filter for relevant changes (*p* < 0.05). For collagen-H (M6) as well as for fibrillar collagen-I and -III (M7,9), it showed a reduction in almost all parameters except for P1 in the presence of Syk-IN (Figure 6B). The parameters of platelet aggregation and contraction (P2,4,5) and platelet activation (P6 for M6, and P7,8, for M7,9) were the most reduced.

To obtain an overall insight into the effect of Syk inhibition, a summative plot was again constructed for each scaled parameter across all collagen microspots (Figure 6C). Importantly, this revealed a highly similar effect of Syk inhibition, as previously seen for the collagen peptides. Summing up the values for M6–9, we noticed a near-complete reduction in P6 (M6, PS exposure), along with strong reductions in P2 (platelet aggregate coverage), P4 (thrombus multilayer), P5 (thrombus contraction), P7 (CD62P expression), and P8 (fibrinogen binding) compared to vehicle-treated blood. Less affected by Syk inhibition was P3 (thrombus morphology), while platelet adhesion (P1) was unchanged.

In view of a possible role of GPVI also for platelet interaction with collagen type IV, additional whole-blood flow runs (*n* = 3 donors) were performed with collagen-IV microspots. Under control conditions, we noticed a pattern of thrombus formation resembling that of collagen-I (M7) or collagen-III (M9). In the presence of Syk-IN, all parameters on collagen-IV were significantly reduced (*p* < 0.01), with the exception of P2 and P6. Across all parameters, the median inhibitory effect of Syk-IN for collagen-I, -III, and -IV was 87.8%, 88.0%, and 85.7%, respectively (data not shown). Hence, we observed a similar extent of thrombus inhibition by Syk-IN for all of these fibrillar collagens.

### 2.5. Modeling of the Role of GPVI in Thrombus Formation on Various Collagens

We then applied a regression analysis to provide a systematic examination of the generated data (M1–9), which consisted of 416 data points (52 mean control flow runs of 9 surfaces, 8 parameters), to reveal the GPVI dependency of each surface. First, a partial least square (PLS) regression model was generated for collagen peptides M1–3 (which had an assumed high GPVI dependency) and for M4,5 (with supposedly no role for GPVI), after which the data from M6 (collagen-H) were entered into the model. This analysis resulted in relevant components 1 and 2, explaining 68% and 15% of the variation, respectively (Figure S4). A principal component plot indicated a tight cluster of flow runs with M1–3,6. The data for M5 (negative component 1) and M4 (negative component 2) lay further out in the model. This agreed with the large observed differences in (the parameters of) thrombus formation on M4 and M5. The calculated beta matrix indicated that P2–6 contributed to the modeled results to a similar extent.

Because of the separation of M4–5 parameters, the component 1 model was used for further analysis. Prediction testing of the model showed near complete prediction accuracy for all surfaces, except for M4 (because there was no component 2) (Table 2). The model was further used to predict the role of GPVI in the remaining collagen surfaces, M7–9. For both fibrillar collagens (M7,9), the prediction of GPVI dependency was mixed, while it was negative for the degraded collagen-I (M8). Subsequently, we integrated into the model the second set of 416 data points of Syk-inhibited blood samples (52 mean flow runs with Syk-IN for 9 surfaces, 8 parameters) to predict the absence of GPVI activity. Markedly, across all surfaces, 51 out of 52 samples predicted a negative GPVI dependency, wherein the only incorrectly predicted sample was just above the conventional 0.5 threshold value for right prediction. Taken together, the constructed PLS model indicated, in addition to complete GPVI-independency of all Syk-inhibited samples, no role of GPVI on surfaces M5 and M8.

## 3. Discussion

### 3.1. Collagen Peptides and GPVI-Dependent Platelet Activation

The data obtained indicated a clear separation between the effects of triple-helical collagen peptides that contained the established GPVI recognition motif, (GPO)_n_ [15], and peptides that had a (GPP)_n_ backbone instead. We found that the (GPO)_n_-containing collagen peptides (M1–3) (i) induced high platelet Ca^2+^ rises under stasis, (ii) accomplished a fast build-up of thrombi with aggregated and activated platelets under flow, and (iii) evoked platelet responses both under flow and static conditions that were highly sensitive to the inhibition of Syk. Accordingly, these peptides provided strong proof-of-principle evidence for potent stimulation of the GPVI-Syk-PLCγ2-Ca^2+^ pathway of platelet activation.

Immobilized, the (GPO)_n_ peptide CRP-XL (M2), lacking an α_2_β_1_ interaction motif, produced smaller-sized thrombi (low parameter values in P2–6) than the other collagen peptides did, which was in agreement with the known synergy between GPVI, integrin α_2_β_1_, and GPIb-V-IX receptors in thrombus formation at a high shear rate [9,10,11]. Synergy of GPVI and α_2_β_1_ could also explain why peptides containing the integrin-binding motif G(F/A)OGER evoked a faster Ca^2+^ signal when compared to CRP-XL. Seemingly in contrast with its lower binding affinity to platelets under stasis [17], we observed higher parameters of thrombus formation with GAOGER-GPO (M3) than with GFOGER-GPO (M1). The explanation for this higher activity remains unclear.

In contrast, the (GPP)_n_-containing peptides GFOGER-GPP (M4) and VWF-BP (M5) did not evoke detectable Ca^2+^ rises in platelets under stasis. Yet, when immobilized under flow, the integrin-binding peptide GFOGER-GPP evoked low-parameter thrombus formation in terms of platelet activation and aggregation, and this activity was again suppressed by Syk inhibition. This may have reflected weak interaction of the (GPP)_n_ motif with GPVI, reinforced by strong integrin-binding activity.

Jointly, these results pointed to a Syk-dependent role via GPVI in the support of thrombus formation. This conclusion was supported by a reanalysis of earlier experiments, where effects of the single-chain variable fragment antibody 10B12 were studied for the surfaces M1, M5, and M6 [16]. Markedly, image reanalysis providing the parameters P1,3–6 indicated a similar effect pattern of 10B12 as presently seen with Syk-IN (not shown).

### 3.2. Collagens and GPVI-Dependent Platelet Activation

Fibrillar type I and type III collagens are among the vessel wall components that most strongly activate platelets [7,30]. Due to the structural complexity of multiple adjacent triple helices in these collagens, little is known about how platelet receptors bind to the fibrils, although there is evidence that the copresence of multiple binding sites in a collagen fiber enforces platelet adhesion and activation [31,32]. Recent high-resolution microscopy has indicated that multiple copies of GPVI dimerize and cluster along the fibers of such collagens, a process that is considered to enforce GPVI-dependent platelet activation [33,34]. Previous sequence analysis has shown that both type I and III collagens are made for up to 10% of GPO triplets, with α_2_β_1_ binding sequences present in both cases, e.g., GFOGER in collagen-I and GAOGER in collagen-III [35].

Here, we compared the effects of preparations of human fibrillar collagen-I and collagen-III to the standard collagen-H, i.e., a commercial equine type I-enriched preparation with fewer defined supramolecular characteristics [36]. Markedly, added to suspended platelets, collagen-H (M6) was the only collagen that induced Syk-dependent [Ca^2+^]_i_ rises, whereas the other collagens (M7,9) induced low [Ca^2+^]_i_ rises that were insensitive to Syk inhibition. When microspotted, collagen-H triggered the formation of large-size thrombi, with high parameters of platelet aggregation and activation, i.e., responses that are known to be strongly GPVI-dependent [9] and that in the present setting were consistently affected by Syk inhibition.

In addition, we tested a protease-treated, monomeric collagen-I preparation (M8), which appeared to be inactive in supporting thrombus formation with no appreciable effect of Syk inhibition. This finding supports the notion that the fibrillar structure of immobilized collagens helps to expose receptor (GPVI) binding sites upon stretching under flow conditions.

In comparison to collagen-H, the immobilized type I (M7) and type III (M9) collagens triggered the formation of smaller thrombi with lower platelet activation parameters. Yet, for the fibrillar collagens, the summed suppressive effects of Syk inhibition were remarkably similar to those seen for collagen-H and the (GPO)_n_-containing collagen peptides. Given the similar abundance of GPO triplets in both collagen-I and -III [15], these findings point to a limited role of GPVI-induced activation under flow conditions. In agreement with such a role for GPVI, others have shown that immobilized collagens can induce GPVI dimer clustering in adhered and spreading platelets [34]. In this setting, immobilized collagen-III was found to be more effective in cluster formation than collagen-H or CRP-XL. Furthermore, the inhibition of Syk did not abrogate the GPVI clustering. These findings suggest that there is not a direct link between GPVI cluster formation and the strength of the GPVI-Syk-PLCγ2 signal. However, under flow conditions, the additional involvement of VWF/GPIb-V-IX and integrin α_2_β_1_ interactions [13,16] might enforce the GPVI clustering pattern, but this still needs to be demonstrated.

### 3.3. Comparative Roles of GPVI and Syk in Platelet Activation

A remarkable finding was that Syk inhibition also affected parameters of thrombus formation on surfaces that were considered to act independently of GPVI (i.e., GFOGER-GPP, M4) or with a low GPVI dependency (collagen-I, M7; collagen-III, M9). As another approach to examine this, a PLS model was constructed and used for principal component analysis. The PCA plots indicated a narrow cluster for all high GPVI-activating surfaces (M1–3,6), with the data of M4,5 partly centering out. A prediction of the role of GPVI for other surfaces gave a mixed outcome for the fibrillar collagens (M7,9), whereas this was negative for the degraded collagen-I (M8). Importantly, the prediction model revealed a consistent GPVI independence for the Syk-inhibited samples, regardless of the type of microspot composition. Accordingly, this analysis supported the indication of low-level GPVI and Syk activity at these weakly thrombogenic surfaces.

In recent years, evidence has accumulated on the role of GPVI signaling in platelets in (also) contacting non collagen surfaces. For instance, GPVI dependency has been discovered for platelets interacting with laminin [37], fibrin [29,38], or fibrinogen [39,40]. In this context, it is also likely that for (GPP)_n_-containing surfaces, Syk-dependent platelet responses can be traced back to residual GPVI activity. On the other hand, based on early studies, it cannot yet be excluded that (part of) the Syk-dependent platelet responses in thrombus formation at “weaker” surfaces are mediated by signaling via integrin α_IIb_β_3_ [41,42,43], hence bypassing GPVI. This needs to be studied using specific GPVI-inhibitory tools.

### 3.4. Conclusion

**Figure 7:**
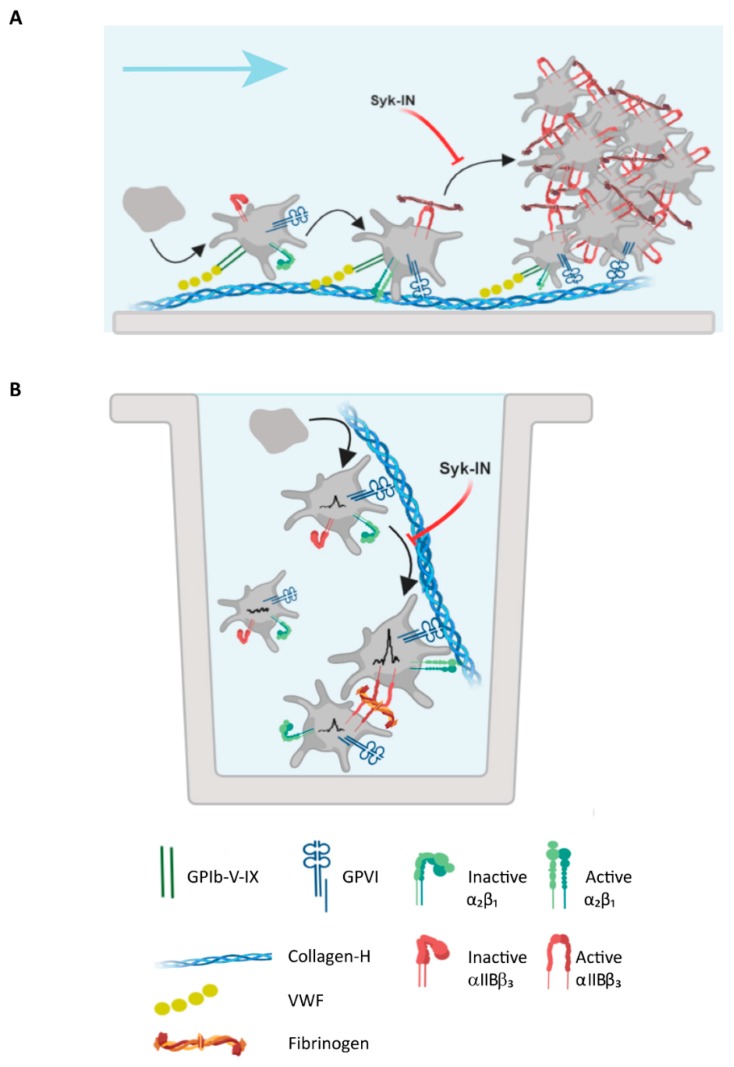
Schematic platelet adhesion and activation by collagen under flow or in suspension. (**A**) Under flow conditions, immobilized collagen-H interacted with VWF to capture platelets via GPIb-V-IX and activate platelets via GPVI and integrin α_2_β_1_. Thrombi built up through the recruitment of flowing platelets interacting with collagen/VWF-adhered platelets. Syk inhibition suppressed initial platelet activation and platelet aggregate formation. (**B**) Collagen-H added to a suspension of platelets transiently interacted with GPVI, resulting in Syk-dependent Ca^2+^ rises. Autocrine agonists stimulated non-adhered platelets, responding through Syk-independent signals.

The present data revealed typical differences in the preparations of collagens or collagen peptides if used in suspension with platelets or when immobilized as microspots and subjected to whole-blood flow (Figure 7). Especially for the “weaker” fibrillar collagens, immobilization appeared to enhance the signaling capability of GPVI, thus stimulating Syk-dependent platelet activation processes in thrombus formation. Apart from changes in the (immobilized) collagen structure, other factors that may have contributed to enhanced signaling capacity were the shear-dependent interaction of GPIb-V-IX with collagen-bound VWF and the priming of platelet activation via integrin α_2_β_1_. These, and perhaps also other receptor interactions with collagen fibers, may have ensured increased activation of the GPVI-PLCγ2-Ca^2+^ pathway.

## 4. Materials and Methods

### 4.1. Materials

Collagen-related triple-helical peptides were synthesized as C-terminal amides and purified by reverse-phase high-performance liquid chromatography [44,45]: H-GPC(GPO)_3_GFO GER(GPO)_3_GPC-NH_2_ (GFOGER-GPO), H-GPC(GPP)_5_GFOGER(GPP)_5_GPC-NH_2_ (GFOGER-GPP), cross-linked collagen-related (GPO)_n_ peptide (CRP-XL), GPC(GPO)_3_GAOGER(GPO)_3_GPC-NH_2_ (GAOGER-GPO), collagen type III-derived VWF-binding peptide VWF-III (VWF-BP), H-GPC(GPP)_5_GPRGQOGVMGFO(GPP)_5_GPC-NH_2_ [46]. Collagen-I Horm derived from equine tendon (collagen-H) was obtained from Nycomed (Hoofddorp, the Netherlands). Human placenta-derived collagen-III (C4407), collagen-IV (C7521), and fibrillar collagen-I (C7774) came from Sigma-Aldrich (Zwijndrecht, the Netherlands). The latter was used to prepare monomeric collagen-I through pepsin treatment, as described in Reference [47]. The selective spleen tyrosine kinase (Syk) inhibitor PRT-060318, 2-((1*R*,2*S*)-2-aminocyclohexylamino)-4-(*m*-tolylamino)pyrimidine-5-carboxamide (Syk-IN), came from Bio-Connect (Huissen, the Netherlands). Used for fluorescence staining were Alexa Fluor (AF)647-labeled antihuman CD62P mAb (304918, Biolegend, London, UK), FITC-labeled fibrinogen (F0111, Dako, Amstelveen, the Netherlands), and AF568-labeled annexin A5 (A13202, ThermoFisher, Eindhoven, the Netherlands). Fura-2 acetoxymethyl ester and pluronic were from Invitrogen (Carlsbad, CA, USA). Human α−thrombin was from Kordia (Leiden, the Netherlands). Stable ADP (Me-S-ADP) and MRS-2179 were from Sigma-Aldrich. Other materials were from sources described before [48].

### 4.2. Blood Isolation

Blood was obtained through venipuncture from healthy volunteers who had not received antiplatelet medication for at least two weeks. All subjects gave their full informed consent according to the Declaration of Helsinki. Studies were approved by the local Medical Ethics Committee. Blood samples were collected into 3.2% trisodium citrate (Vacuette tubes, Greiner Bio-One, Alphen a/d Rijn, the Netherlands). All subjects had platelet counts within the reference range, as measured with a Sysmex XN-9000 analyzer (Sysmex, Cho-ku, Kobe, Japan).

### 4.3. Platelet Isolation and Loading with Fura-2

Platelet-rich plasma (PRP) was obtained from citrated blood through centrifugation at 870 g for 10 min. After the addition of 1:10 vol/vol acid citrate dextrose (ACD; 80 mM trisodium citrate, 183 mM glucose, 52 mM citric acid), the isolated PRP was centrifuged at 2360 g for 2 min. Platelet pellets were resuspended into HEPES buffer, pH 6.6 (10 mM HEPES, 136 mM NaCl, 2.7 mM KCl, 2 mM MgCl_2_, 5.5 mM glucose, and 0.1% bovine serum albumin). After the addition of apyrase (1 U/mL) and 1:15 *v/v* ACD, another centrifugation step was performed to obtain washed platelets [48]. The final pellet was resuspended in HEPES buffer, pH 7.45.

### 4.4. Light Transmission Aggregometry

The aggregation of washed platelets was measured by light transmission aggregometry, as described in Reference [48], using an automated Chronolog aggregometer (Havertown, PA, USA). The platelet aggregation rate was determined from maximal curve slopes (% transmission change per min).

### 4.5. Whole-Blood Microfluidic Perfusion Over Microspots

Selected collagen-like peptides and collagens were microspotted on glass coverslips, essentially as described before [9]. The coding of nine microspots (M1–9) is displayed in Table 1. In brief, washed coverslips were coated with three different microspots, each containing a collagen (100 µg/mL) or a combination of a collagen-like peptide (250 µg/mL) and VWF-BP (100 µg/mL). Coating doses were chosen to obtain maximal platelet adhesion in flow assays [9]. The most active microspots were always located downstream, thus preventing the cross-activation of platelets between microspots [9]. The coated coverslips were incubated overnight in a humid chamber at 4 °C and then blocked with HEPES buffer (pH 7.45) containing 1% bovine serum albumin for 30 min before being mounted into Maastricht microfluidic chambers.

For flow perfusion, 500 µL of citrated whole blood was pre-incubated for 10 min with either vehicle (0.5% DMSO and 0.4 µg/mL pluronic, f.c.) or inhibitor PRT-060318 (Syk-IN, 20 µM in vehicle solution, f.c.). After the addition of 40 µM PPACK and recalcification (3.75 mM MgCl_2_ and 7.5 mM CaCl_2_), blood samples were perfused through microspot-containing flow chambers for 3.5 min at a wall-shear rate of 1000 s^−1^. After 2 min of staining for PS exposure (AF568-annexin A5), CD62P expression (AF647 anti-CD62P mAb), and integrin α_IIb_β_3_ activation (FITC fibrinogen), the residual label was removed by postperfusion with HEPES buffer (pH 7.45) containing 2 mM CaCl_2_ and 1 U/mL heparin. Vehicle controls were performed in duplicate, while samples containing Syk-IN were repeated in triplicate using blood obtained from >5 different healthy donors.

### 4.6. Bright-Field and Fluorescence Microscopy

From each microspot, two bright-field images (during labeling) and three 3-colour fluorescence images (after removing the label) were taken using an EVOS-FL microscope (Life Technologies, Bleiswijk, the Netherlands) equipped with Cy5, RFP, and GFP LEDs; an Olympus UPLSAPO 60× oil immersion objective; and a sensitive 1360 × 1024 pixel CCD camera [49]. A standardized image analysis was performed using semiautomated scripts operated in Fiji (ImageJ), as described before [49]. Parameters extracted from bright-field images (P1–5), including thrombus signature scores (P3–5), and parameters from fluorescence images (P6–8) are specified in Table 1.

### 4.7. Cytosolic Ca^2+^ Measurements

Washed human platelets (2 × 10^8^/mL) were loaded with Fura-2 acetoxymethyl ester (3 μM) and pluronic (0.4 µg/mL) through a 40-min incubation at room temperature. After another wash step and resuspension of the platelets at the same concentration, changes in cytosolic [Ca^2+^]_i_ were measured in 96-well plates using a FlexStation 3 (Molecular Devices, San Jose, CA, USA). In brief, 200 µL of platelet suspension were pretreated with Syk-IN (5 μM) for 10 min or were left untreated. After the addition of 1 mM CaCl_2_, the Fura-2-loaded cells were stimulated by automated pipetting with one of the following agonists (10 μg/mL) (for convenience, indicated as M1–9 (see Table 1)): GFOGER-GPO (M1), CRP-XL (M2), GAOGER-GPO (M3), GFOGER-GPP (M4), VWF-BP (M5), collagen-H (M6), fibrillar collagen-I (M7), monomeric collagen-I (M8), or collagen-III (M9).

Changes in Fura-2 fluorescence were measured over time at 37 °C by ratiometric fluorometry at dual excitation wavelengths of 340 and 380 nm and an emission wavelength of 510 nm. The agonist injection speed was set at 125 μL/s, resulting in complete, diffusion-limited mixing. Separate wells contained Fura-2-loaded platelets that were lysed with 0.1% Triton-X-100 in the presence of either 1 mM CaCl_2_ or 9 mM EGTA/Tris for a determination of the *R_max_* and *R_min_* values, respectively [50]. After correction for background fluorescence, [Ca^2+^]_i_ (as nM) was calculated from the ratio values [51]. Measurements were performed in duplicate wells and were completed within 2–3 h of preparation of the cells.

### 4.8. Data Handling and Statistics

GraphPad Prism 8 was employed for statistical analysis. Heatmaps were generated with the program R. For heatmap representation, all parameter values were univariate-normalized at a scale of 0–10 [46]. Thrombus values of duplicate or triplicate flow runs from the same blood donor were averaged to obtain one parameter set (vehicle or Syk-inhibited) per microspot and donor. Mean values of control and inhibitor runs were then compared per blood sample using paired Student’s *t*-tests: *p*-values below 0.05 were considered to be significant. For subtraction heatmaps, a standard filter of *p* < 0.05 was set to visualize relevant effects.

### 4.9. Modeling to Predict GPVI Activity

Complete datasets (8 parameters, 9 surfaces) for flow runs of ≥5 donors were used to construct a partial least square (PLS) model to predict GPVI dependency. First, range-scaled data for the collagen peptide surfaces (M1–5) with known GPVI dependency were used to generate a PLS model, after which collagen-H (M6) was then used to test the reliability of the model. A principal component analysis (PCA) in 1- and 2-component mode was then applied, the predictions of which were supported by cross-validated analysis of *Q*^2^, defined as 1 - (PRESS/TSS) [52]. Subsequently, parameter sets of M7–9 were predicted for GPVI-dependency from the PLS model, as were parameters of M1–9 in the presence of Syk-IN. By default, prediction values >0.5 were considered to be positive for GPVI dependency.

## Figures and Tables

**Table 1:** Overview of composition of microspots (M1–9), platelet receptors implicated in thrombus formation. Also indicated are the analyzed thrombus parameters (P1–8) from bright-field and fluorescence microscopic images. Measured ranges and scaling for heatmap analysis were as indicated. GP: Glycoprotein; PS: Phosphatidylserine; VWF-BP: von Willebrand factor binding peptide, SAC: Surface area coverage, n.a., not assessed.

**Microspot**		Platelet Receptors		
		*GPVI*	*α_2_β_1_*	*GPIb*
M1	GFOGER-GPO + VWF-BP	++	++	++
M2	CRP-XL + VWF-BP	++	o	++
M3	GAOGER-GPO + VWF-BP	++	+	++
M4	GFOGER-GPP + VWF-BP	(o)*	++	++
M5	VWF-BP	o	o	++
M6	collagen-H (Horm type)	++	++	++
M7	collagen-I (human)	n.a.	n.a.	++
M8	monomeric collagen-I (human)	n.a.	n.a.	++
M9	collagen-III (human)	n.a.	n.a.	++
**Parameter**		range	scaling	
*Bright-Field Images*				
P1	**platelet deposition (% SAC)**	0–51.52	0–10	
P2	platelet aggregate coverage (% SAC)	0–21.09	0–10	
P3	thrombus morphological score	0–4.10	0–10	
P4	thrombus multilayer score	0–2.60	0–10	
P5	thrombus contraction score	0–2.94	0–10	
*Fluorescence Images*				
P6	PS exposure (% SAC)	0–13.91	0–10	
P7	CD62P expression (% SAC)	0–46.71	0–10	
P8	fibrinogen binding (% SAC)	0–28.33	0–10	

* No GPVI-activating (GPP)_n_ motif.

**Table 2:** Modeled partial least square (PLS) analysis (based on component 1 principal component analysis (PCA)) of range-scaled data for collagen peptides (M1–5) plus collagen-H (M6), with assumed GPVI dependency. The ranges of prediction values are shown. Predicted accuracy is given by numbers of mean flow runs per donor (control and Syk-IN). By default, a correct prediction was set at >0.5. In addition, back-prediction of GPVI dependency of mean flow runs per donor for M7–9 is shown. Prediction outcomes are given here in italics. The contributions of parameters to the prediction model were in the order of P2–6 >> P1,7,8.

Microspot	GPVI Dependency	Correctly Predicted		
		Range	Ctrl	Syk-IN
M1	positive	0.41–1.06	5/6	6/6
M2	positive	0.27–0.76	4/5	5/5
M3	positive	0.86–1.07	5/5	5/5
M4	negative	0.57–0.97	0/6	6/6
M5	negative	0.21–0.34	5/5	5/5
M6	positive	0.68–1.11	7/7	6/7
M7	mixed	0.44–0.85	5/7	7/7
M8	negative	0.13–0.41	0/5	5/5
M9	mixed	0.49–0.67	3/5	5/5

## Supplementary Materials

Supplementary materials can be found at https://www.mdpi.com/1422-0067/20/11/2788/s1. See separate file.
