# Supplementary material for: Role of Platelet Glycoprotein VI and Tyrosine Kinase Syk in Thrombus Formation on Collagen-Like Surfaces
Source: Int J Mol Sci. 2019 Jun 7;20(11):2788. doi: 10.3390/ijms20112788 (PMC6600290; doi:10.3390/ijms20112788)
Supplement: Supplementary file 1 [file ijms-20-02788-s001.pdf]

Supplementary Materials

Role of platelet glycoprotein VI and tyrosine kinase Syk in thrombus formation on collagen-like surfaces

Natalie J. Jooss,[1*] Ilaria De Simone,[1*] Isabella Provenzale,[1*] Delia I. Fernandez,[1*] Sanne L. N. Brouns,[1] Richard W. Farndale,[2] Yvonne M. C. Henskens,[3] Marijke J. E. Kuijpers,[1] Hugo ten Cate,[1,5] Paola E. J. van der Meijden,[1*] Rachel Cavill,[4*] Johan W. M. Heemskerk[1]†

Departments of Biochemistry[1] and Data Science and Knowledge Engineering[4], Cardiovascular Research Institute, Maastricht University; Central Diagnostic Laboratory[3] and Department of Internal Medicine[5], Maastricht University Medical Centre, Maastricht, The Netherlands. Department of Biochemistry[2], University of Cambridge, Cambridge, United Kingdom

* Equal contribution

† Correspondence: jwm.heemskerk@maastrichtuniversity.nl; Tel.: +31-43-3881671 (3881674) (NL)

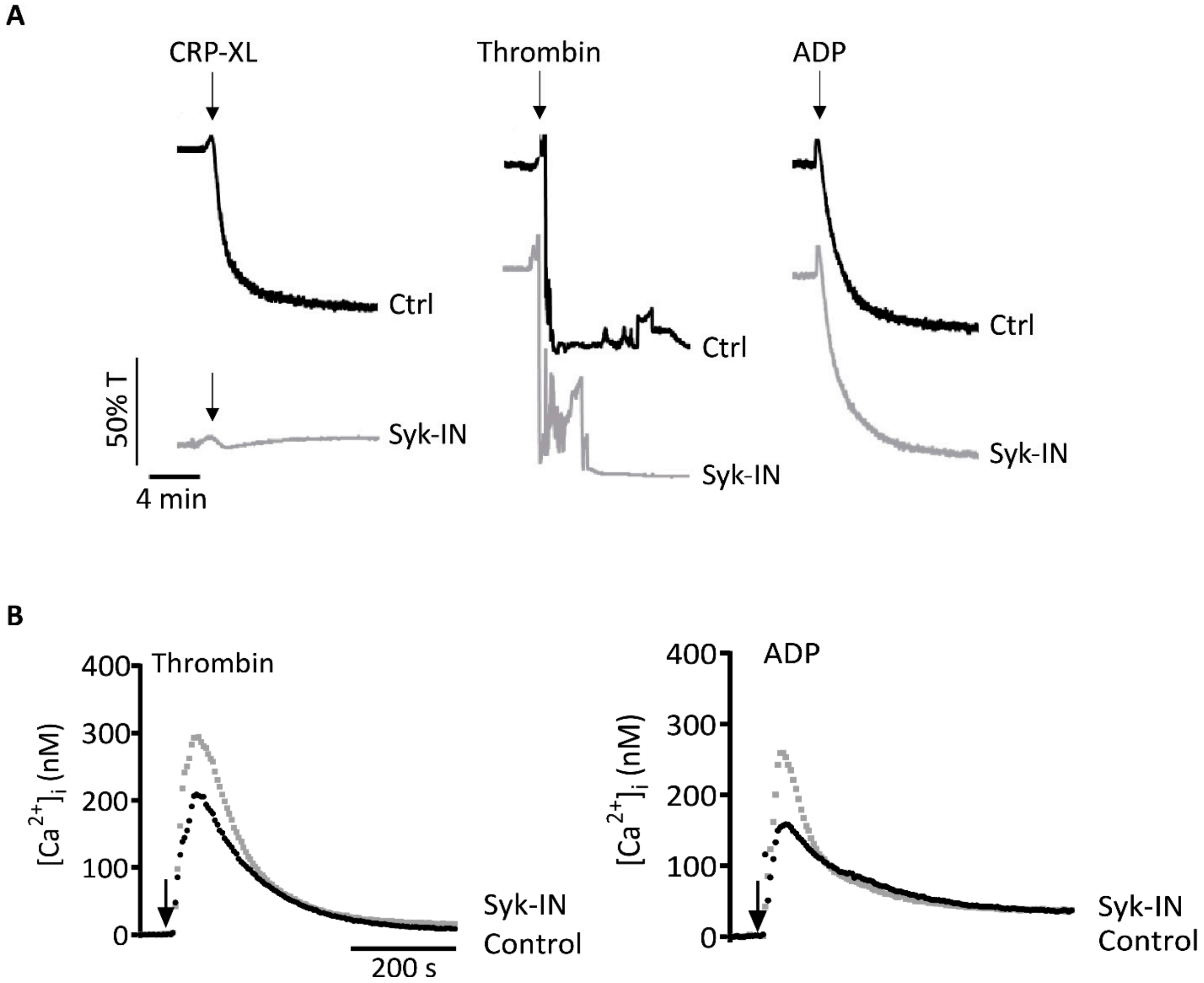

FIGURE S1. Effect of Syk inhibitor PRT-060318 (Syk-IN) on agonist-induced platelet responses. **(A)** Platelets in plasma (2.5 x 10^8/ml) were pre-incubated with vehicle (DMSO) or Syk-IN (5 μM) for 10 min, and then activated with CRP-XL (10 μg/ml), thrombin (8 nM) or stable ADP (5 μM), as indicated. Shown are representative traces from light transmission aggregometry. **(B)** Fura-2-loaded platelets in 96-well plates were pre-incubated with Syk-IN (5 μM) or left untreated before injection of thrombin (4 nM) or stable ADP (5 μM), as in Figure 1. Shown are representative traces of changes in $[Ca^{2+}]_i$ of control (black) and Syk-IN (grey) incubations. Arrows indicate addition indicated of agonists.

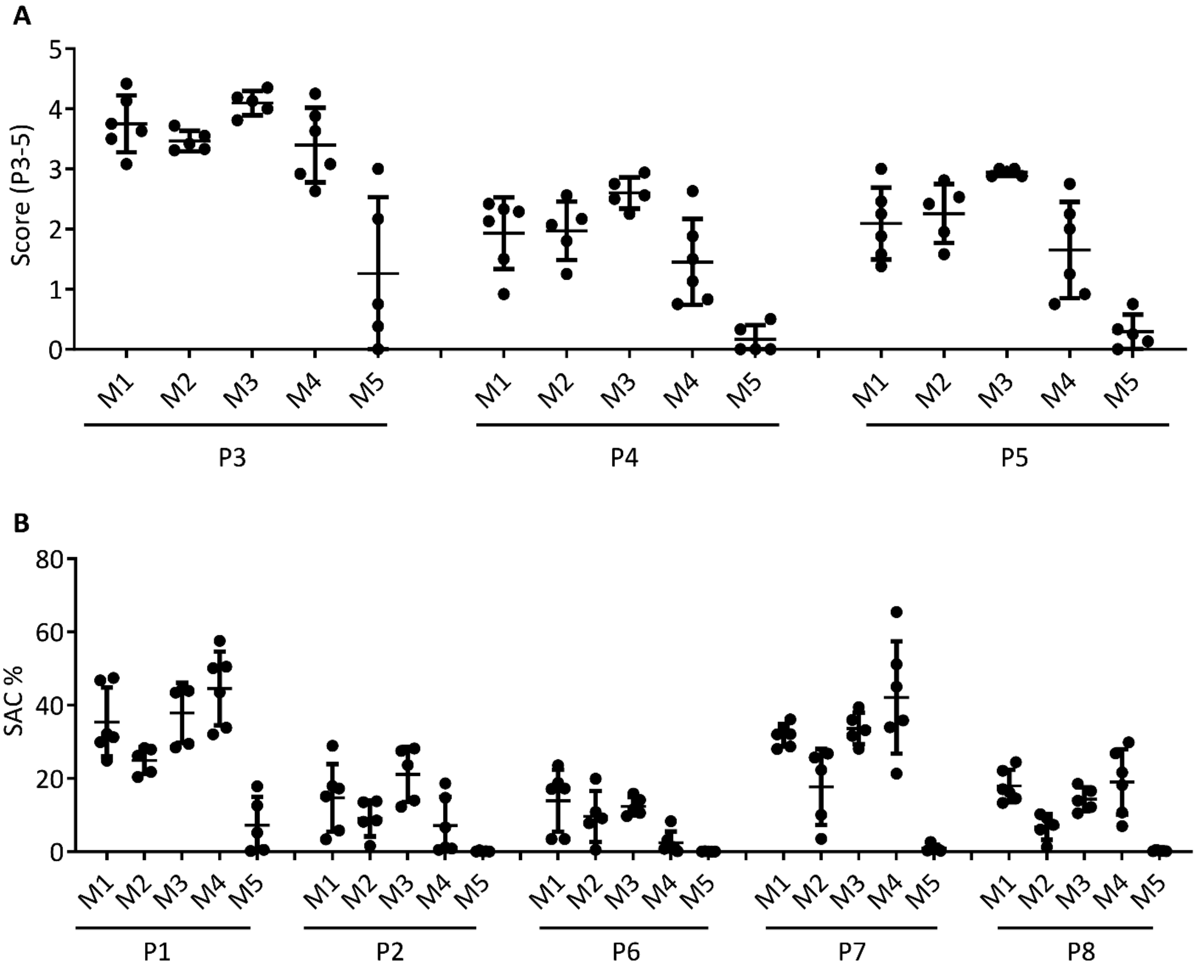

FIGURE S2. Parameters of thrombus formation on immobilised collagen peptides: raw data. Whole-blood was perfused over microspots *M1* (GFOGER-GPO + VWF-BP), *M2* (CRP-XL + VWF-BP), *M3* (GAOGER-GPO + VWF-BP), *M4* (GFOGER-GPP + VWF-BP), and *M5* (VWF-BP). Microscopic images were analysed for parameters *P1-8*, as for Figure 2. Shown are raw mean outcome values from individual blood donors. **(A)** Parameters providing surface area coverage (SAC%) information: *P1*, platelet deposition; *P2*, platelet aggregate coverage; *P6*, PS exposure; *P7*, CD62P expression; *P8*, fibrinogen binding. **(B)** Score parameters: *P3*, thrombus morphological score (range 0-5); *P4*, thrombus multilayer score (range 0-3); *P5*, thrombus contraction score (range 0-3). Means ± SD (n=5-7 donors).

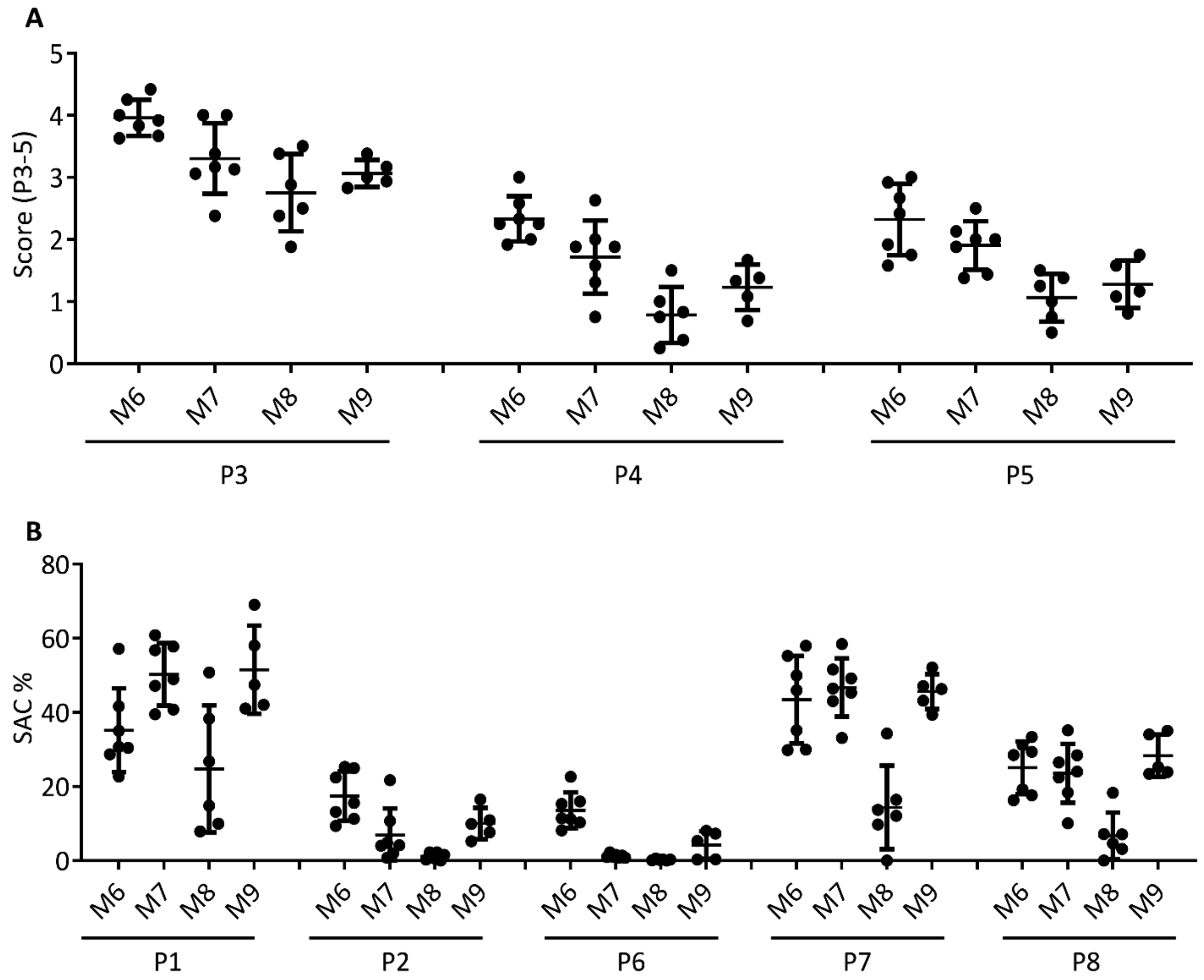

FIGURE S3. Parameters of thrombus formation on immobilised collagens: raw data. Whole-blood was perfused over microspots *M6* (collagen-H), *M7* (fibrillar collagen-I), *M8* (monomeric collagen-I), *M9* (collagen-III). Microscopic images were captured and analysed for parameters P1-8, as for Figure 5. Shown are raw mean outcome values from individual blood donors. **(A)** Parameters providing surface area coverage (SAC%) information. **(B)** Score parameters. See further Suppl. Figure S2. Means ± SD (n=5-7 donors).

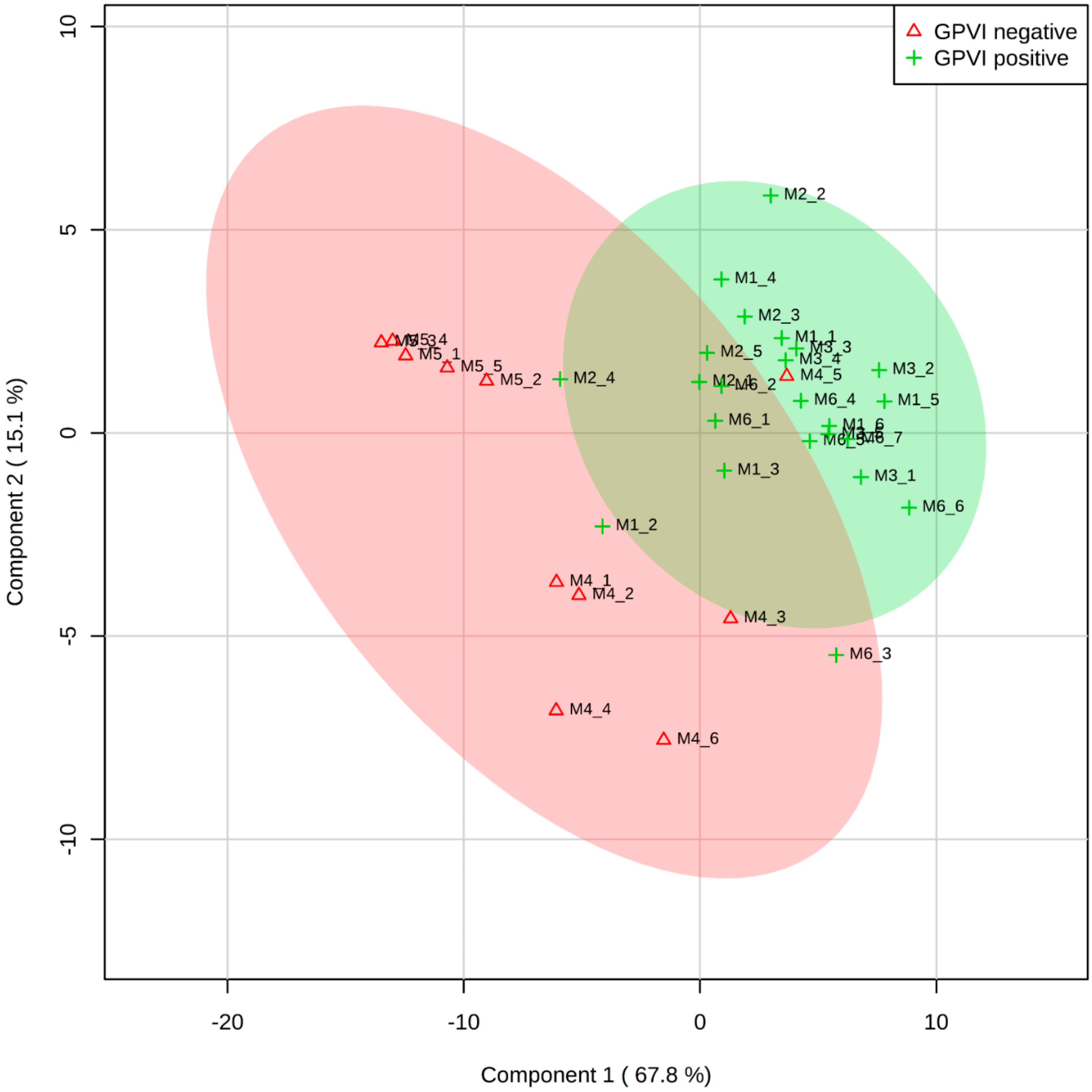

FIGURE S4. Partial Least Squares with components 1 and 2, indicating distribution of thrombus formation parameters at microspots *M1-6* for 5-7 individual blood samples per microspot. Note, the clustering (green area) of flow runs over $(GPO)_n$ containing surfaces *M1-3* and *M6*, whereas flow runs with other surfaces M4 and M5 out-clustered with more negative contributions to component 2 or 1, respectively (red area). Red triangles indicate assumed negative GPVI contribution, green plusses indicate positive contribution.

TABLE S1. Scaled subtracted parameter values of thrombus formation (means), indicating effects of Syk-IN, for microspots *M1-9* and parameters *P1-8*.

	P1	*P2*	*P3*	*P4*	*P5*	*P6*	*P7*	*P8*
M1	1.18±0.46	0.10±0.14	0.51±0.17	0.15±0.16	0.21±0.20	0.03±0.05	0.71±0.49	0.62±0.66
M2	1.15±0.24	0.16±0.13	0.77±0.15	0.30±0.20	0.30±0.15	0.04±0.06	0.69±0.39	0.31±0.28
M3	0.94±0.24	0.10±0.07	0.71±0.17	0.37±0.23	0.34±0.12	0.01±0.00	0.47±0.27	0.11±0.08
M4	0.93±0.29	0.17±0.29	0.73±0.09	0.34±0.34	0.37±0.40	0.13±0.16	0.25±0.18	0.04±0.03
M5	0.97±1.18	1.27±2.13	0.13±0.12	0.00±0.00	0.00±0.00	0.63±0.79	1.40±2.65	0.36±0.43
M6	1.12±0.33	0.19±0.10	0.61±0.15	0.22±0.17	0.28±0.18	0.02±0.02	0.65±0.35	0.42±0.44
M7	0.88±0.21	0.03±0.06	0.60±0.20	0.10±0.11	0.17±0.12	0.03±0.02	0.13±0.08	0.02±0.02
M8	0.87±0.56	2.91±6.23	0.61±0.35	0.64±0.74	0.42±0.28	0.16±0.15	0.25±0.16	0.12±0.17
M9	0.79±0.20	0.07±0.07	0.49±0.06	0.13±0.23	0.11±0.13	0.03±0.03	0.14±0.12	0.05±0.05
